# Supplementary material for: Identification of the Genetic Basis of Response to de-Acclimation in Winter Barley
Source: Int J Mol Sci. 2021 Jan 21;22(3):1057. doi: 10.3390/ijms22031057 (PMC7865787; doi:10.3390/ijms22031057)
Supplement: Supplementary file 1 [file ijms-22-01057-s001.zip › supplementary/table S1.docx]

**Table S1.** Significant (FDR<0.05) GO terms for differentially expressed genes specific only to de-acclimation.

| **GO term** | **Ontology** | **Description** | **Number of DEGs and direction of regulation referred to cold-acclimated state; T - Tolerant, S - susceptible** |
| --- | --- | --- | --- |
| GO:0019438 | Biological Process | aromatic compound biosynthetic process | 16 down-regulated (S) |
| GO:0006754 | Biological Process | ATP biosynthetic process | 8 down-regulated (S) |
| GO:0046034 | Biological Process | ATP metabolic process | 9 down-regulated (S) |
| GO:0015986 | Biological Process | ATP synthesis coupled proton transport | 8 down-regulated (S) |
| GO:0009058 | Biological Process | biosynthetic process | 22 down-regulated (S) |
| GO:1901137 | Biological Process | carbohydrate derivative biosynthetic process | 8 down-regulated (S) |
| GO:1901135 | Biological Process | carbohydrate derivative metabolic process | 9 down-regulated (S) |
| GO:0098655 | Biological Process | cation transmembrane transport | 8 down-regulated (S) |
| GO:0006812 | Biological Process | cation transport | 8 down-regulated (S) |
| GO:0008037 | Biological Process | cell recognition | 6 up-regulated (S) |
| GO:0006725 | Biological Process | cellular aromatic compound metabolic process | 23 down-regulated (S) |
| GO:0044249 | Biological Process | cellular biosynthetic process | 21 down-regulated (S) |
| GO:0034641 | Biological Process | cellular nitrogen compound metabolic process | 24 down-regulated (S) |
| GO:0006464 | Biological Process | cellular protein modification process | 44 up-regulated (13 T, 31 S) |
| GO:0015985 | Biological Process | energy coupled proton transport, down electrochemical gradient | 8 down-regulated (S) |
| GO:0051234 | Biological Process | establishment of localization | 40 S (25 up-regulated, 15 down-regulated) |
| GO:1901659 | Biological Process | glycosyl compound biosynthetic process | 8 down-regulated (S) |
| GO:1901657 | Biological Process | glycosyl compound metabolic process | 9 down-regulated (S) |
| GO:0018130 | Biological Process | heterocycle biosynthetic process | 16 down-regulated (S) |
| GO:0046483 | Biological Process | heterocycle metabolic process | 23 down-regulated (S) |
| GO:1902600 | Biological Process | hydrogen ion transmembrane transport | 8 down-regulated (S) |
| GO:0006818 | Biological Process | hydrogen transport | 8 down-regulated (S) |
| GO:0098662 | Biological Process | inorganic cation transmembrane transport | 8 down-regulated (S) |
| GO:0098660 | Biological Process | inorganic ion transmembrane transport | 8 down-regulated (S) |
| GO:0034220 | Biological Process | ion transmembrane transport | 8 down-regulated (S) |
| GO:0006811 | Biological Process | ion transport | 8 down-regulated (S) |
| GO:0051179 | Biological Process | localization | 40 S (25 up-regulated, 15 down-regulated) |
| GO:0043412 | Biological Process | macromolecule modification | 44 up-regulated (13 T, 31 S) |
| GO:0015672 | Biological Process | monovalent inorganic cation transport | 8 down-regulated (S) |
| GO:0032501 | Biological Process | multicellular organismal process | 6 up-regulated (S) |
| GO:0044706 | Biological Process | multi-multicellular organism process | 6 up-regulated (S) |
| GO:0051704 | Biological Process | multi-organism process | 6 up-regulated (S) |
| GO:0044703 | Biological Process | multi-organism reproductive process | 6 up-regulated (S) |
| GO:0006807 | Biological Process | nitrogen compound metabolic process | 27 down-regulated (S) |
| GO:0034654 | Biological Process | nucleobase-containing compound biosynthetic process | 16 down-regulated (S) |
| GO:0006139 | Biological Process | nucleobase-containing compound metabolic process | 23 down-regulated (S) |
| GO:0055086 | Biological Process | nucleobase-containing small molecule metabolic process | 11 down-regulated (S) |
| GO:0009163 | Biological Process | nucleoside biosynthetic process | 8 down-regulated (S) |
| GO:0009116 | Biological Process | nucleoside metabolic process | 9 down-regulated (S) |
| GO:0009124 | Biological Process | nucleoside monophosphate biosynthetic process | 8 down-regulated (S) |
| GO:0009123 | Biological Process | nucleoside monophosphate metabolic process | 9 down-regulated (S) |
| GO:1901293 | Biological Process | nucleoside phosphate biosynthetic process | 9 down-regulated (S) |
| GO:0006753 | Biological Process | nucleoside phosphate metabolic process | 10 down-regulated (S) |
| GO:0009142 | Biological Process | nucleoside triphosphate biosynthetic process | 8 down-regulated (S) |
| GO:0009141 | Biological Process | nucleoside triphosphate metabolic process | 9 down-regulated (S) |
| GO:0009165 | Biological Process | nucleotide biosynthetic process | 9 down-regulated (S) |
| GO:0009117 | Biological Process | nucleotide metabolic process | 10 down-regulated (S) |
| GO:1901362 | Biological Process | organic cyclic compound biosynthetic process | 16 down-regulated (S) |
| GO:1901360 | Biological Process | organic cyclic compound metabolic process | 23 down-regulated (S) |
| GO:1901576 | Biological Process | organic substance biosynthetic process | 21 down-regulated (S) |
| GO:1901566 | Biological Process | organonitrogen compound biosynthetic process | 14 down-regulated (S) |
| GO:1901564 | Biological Process | organonitrogen compound metabolic process | 15 down-regulated (S) |
| GO:0090407 | Biological Process | organophosphate biosynthetic process | 9 down-regulated (S) |
| GO:0019637 | Biological Process | organophosphate metabolic process | 10 down-regulated (S) |
| GO:0006796 | Biological Process | phosphate-containing compound metabolic process | 27 up-regulated (S) |
| GO:0006793 | Biological Process | phosphorus metabolic process | 27 up-regulated (S) |
| GO:0016310 | Biological Process | phosphorylation | 39 up-regulated (12 T, 27 S) |
| GO:0009875 | Biological Process | pollen-pistil interaction | 6 up-regulated (S) |
| GO:0009856 | Biological Process | pollination | 6 up-regulated (S) |
| GO:0036211 | Biological Process | protein modification process | 44 up-regulated (13 T, 31 S) |
| GO:0006468 | Biological Process | protein phosphorylation | 39 up-regulated (12 T, 27 S) |
| GO:0015992 | Biological Process | proton transport | 8 down-regulated (S) |
| GO:0042451 | Biological Process | purine nucleoside biosynthetic process | 8 down-regulated (S) |
| GO:0042278 | Biological Process | purine nucleoside metabolic process | 9 down-regulated (S) |
| GO:0009127 | Biological Process | purine nucleoside monophosphate biosynthetic process | 8 down-regulated (S) |
| GO:0009126 | Biological Process | purine nucleoside monophosphate metabolic process | 9 down-regulated (S) |
| GO:0009145 | Biological Process | purine nucleoside triphosphate biosynthetic process | 8 down-regulated (S) |
| GO:0009144 | Biological Process | purine nucleoside triphosphate metabolic process | 9 down-regulated (S) |
| GO:0006164 | Biological Process | purine nucleotide biosynthetic process | 8 down-regulated (S) |
| GO:0006163 | Biological Process | purine nucleotide metabolic process | 9 down-regulated (S) |
| GO:0046129 | Biological Process | purine ribonucleoside biosynthetic process | 8 down-regulated (S) |
| GO:0046128 | Biological Process | purine ribonucleoside metabolic process | 9 down-regulated (S) |
| GO:0009168 | Biological Process | purine ribonucleoside monophosphate biosynthetic process | 8 down-regulated (S) |
| GO:0009167 | Biological Process | purine ribonucleoside monophosphate metabolic process | 9 down-regulated (S) |
| GO:0009206 | Biological Process | purine ribonucleoside triphosphate biosynthetic process | 8 down-regulated (S) |
| GO:0009205 | Biological Process | purine ribonucleoside triphosphate metabolic process | 9 down-regulated (S) |
| GO:0009152 | Biological Process | purine ribonucleotide biosynthetic process | 8 down-regulated (S) |
| GO:0009150 | Biological Process | purine ribonucleotide metabolic process | 9 down-regulated (S) |
| GO:0072522 | Biological Process | purine-containing compound biosynthetic process | 8 down-regulated (S) |
| GO:0072521 | Biological Process | purine-containing compound metabolic process | 9 down-regulated (S) |
| GO:0048544 | Biological Process | recognition of pollen | 6 up-regulated (S) |
| GO:0000003 | Biological Process | reproduction | 6 up-regulated (S) |
| GO:0022414 | Biological Process | reproductive process | 6 up-regulated (S) |
| GO:0042455 | Biological Process | ribonucleoside biosynthetic process | 8 down-regulated (S) |
| GO:0009119 | Biological Process | ribonucleoside metabolic process | 9 down-regulated (S) |
| GO:0009156 | Biological Process | ribonucleoside monophosphate biosynthetic process | 8 down-regulated (S) |
| GO:0009161 | Biological Process | ribonucleoside monophosphate metabolic process | 9 down-regulated (S) |
| GO:0009201 | Biological Process | ribonucleoside triphosphate biosynthetic process | 8 down-regulated (S) |
| GO:0009199 | Biological Process | ribonucleoside triphosphate metabolic process | 9 down-regulated (S) |
| GO:0009260 | Biological Process | ribonucleotide biosynthetic process | 8 down-regulated (S) |
| GO:0009259 | Biological Process | ribonucleotide metabolic process | 9 down-regulated (S) |
| GO:0046390 | Biological Process | ribose phosphate biosynthetic process | 8 down-regulated (S) |
| GO:0019693 | Biological Process | ribose phosphate metabolic process | 9 down-regulated (S) |
| GO:0044702 | Biological Process | single organism reproductive process | 6 up-regulated (S) |
| GO:0044711 | Biological Process | single-organism biosynthetic process | 14 down-regulated (S) |
| GO:1902578 | Biological Process | single-organism localization | 10 down-regulated (S) |
| GO:0044710 | Biological Process | single-organism metabolic process | 25 down-regulated (S) |
| GO:0044699 | Biological Process | single-organism process | 29 down-regulated (S) |
| GO:0044765 | Biological Process | single-organism transport | 10 down-regulated (S) |
| GO:0044281 | Biological Process | small molecule metabolic process | 15 down-regulated (S) |
| GO:0055085 | Biological Process | transmembrane transport | 12 down-regulated (S) |
| GO:0006810 | Biological Process | transport | 40 S (25 up-regulated, 15 down-regulated) |
| GO:0030554 | Molecular Function | adenyl nucleotide binding | 50 up-regulated (15 T, 35 S) |
| GO:0032559 | Molecular Function | adenyl ribonucleotide binding | 50 up-regulated (15 T, 35 S) |
| GO:0005524 | Molecular Function | ATP binding | 32 up-regulated (S) |
| GO:0097367 | Molecular Function | carbohydrate derivative binding | 52 up-regulated (15 T, 37 S) |
| GO:0003824 | Molecular Function | catalytic activity | 91 up-regulated (S) |
| GO:0008324 | Molecular Function | cation transmembrane transporter activity | 8 down-regulated (S) |
| GO:0020037 | Molecular Function | heme binding | 13 up-regulated (S) |
| GO:0015078 | Molecular Function | hydrogen ion transmembrane transporter activity | 8 down-regulated (S) |
| GO:0022890 | Molecular Function | inorganic cation transmembrane transporter activity | 8 down-regulated (S) |
| GO:0015075 | Molecular Function | ion transmembrane transporter activity | 8 down-regulated (S) |
| GO:0016301 | Molecular Function | kinase activity | 39 up-regulated (12 T, 27 S) |
| GO:0015077 | Molecular Function | monovalent inorganic cation transmembrane transporter activity | 8 down-regulated (S) |
| GO:0001882 | Molecular Function | nucleoside binding | 51 up-regulated (15 T, 36 S) |
| GO:1901265 | Molecular Function | nucleoside phosphate binding | 17 up-regulated (T) |
| GO:0000166 | Molecular Function | nucleotide binding | 17 up-regulated (T) |
| GO:0016773 | Molecular Function | phosphotransferase activity, alcohol group as acceptor | 39 up-regulated (12 T, 27 S) |
| GO:0004672 | Molecular Function | protein kinase activity | 39 up-regulated (12 T, 27 S) |
| GO:0001883 | Molecular Function | purine nucleoside binding | 51 up-regulated (15 T, 36 S) |
| GO:0017076 | Molecular Function | purine nucleotide binding | 51 up-regulated (15 T, 36 S) |
| GO:0032550 | Molecular Function | purine ribonucleoside binding | 51 up-regulated (15 T, 36 S) |
| GO:0035639 | Molecular Function | purine ribonucleoside triphosphate binding | 33 up-regulated (S) |
| GO:0032555 | Molecular Function | purine ribonucleotide binding | 51 up-regulated (15 T, 36 S) |
| GO:0032549 | Molecular Function | ribonucleoside binding | 51 up-regulated (15 T, 36 S) |
| GO:0032553 | Molecular Function | ribonucleotide binding | 52 up-regulated (15 T, 37 S) |
| GO:0036094 | Molecular Function | small molecule binding | 17 up-regulated (T) |
| GO:0022891 | Molecular Function | substrate-specific transmembrane transporter activity | 9 down-regulated (S) |
| GO:0022892 | Molecular Function | substrate-specific transporter activity | 9 down-regulated (S) |
| GO:0046906 | Molecular Function | tetrapyrrole binding | 13 up-regulated (S) |
| GO:0016740 | Molecular Function | transferase activity | 44 up-regulated (S) |
| GO:0016772 | Molecular Function | transferase activity, transferring phosphorus-containing groups | 39 up-regulated (12 T, 27 S) |
| GO:0022857 | Molecular Function | transmembrane transporter activity | 10 down-regulated (S) |
