# Supplementary figures and images for: Identification of the Genetic Basis of Response to de-Acclimation in Winter Barley
Source: Int J Mol Sci. 2021 Jan 21;22(3):1057. doi: 10.3390/ijms22031057 (PMC7865787; doi:10.3390/ijms22031057)

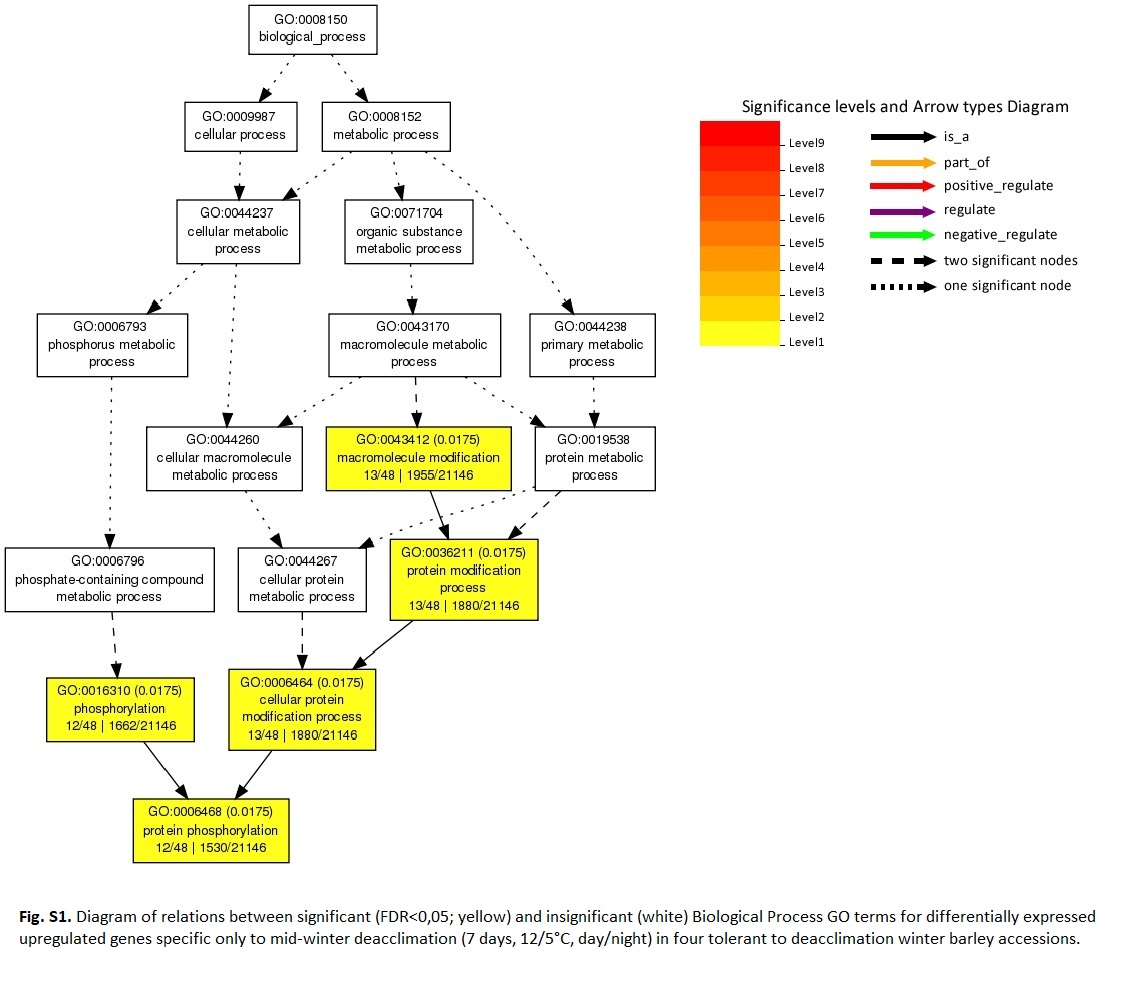

Supplement: Supplementary file 1 [file ijms-22-01057-s001.zip › supplementary/Figure S1.jpg]

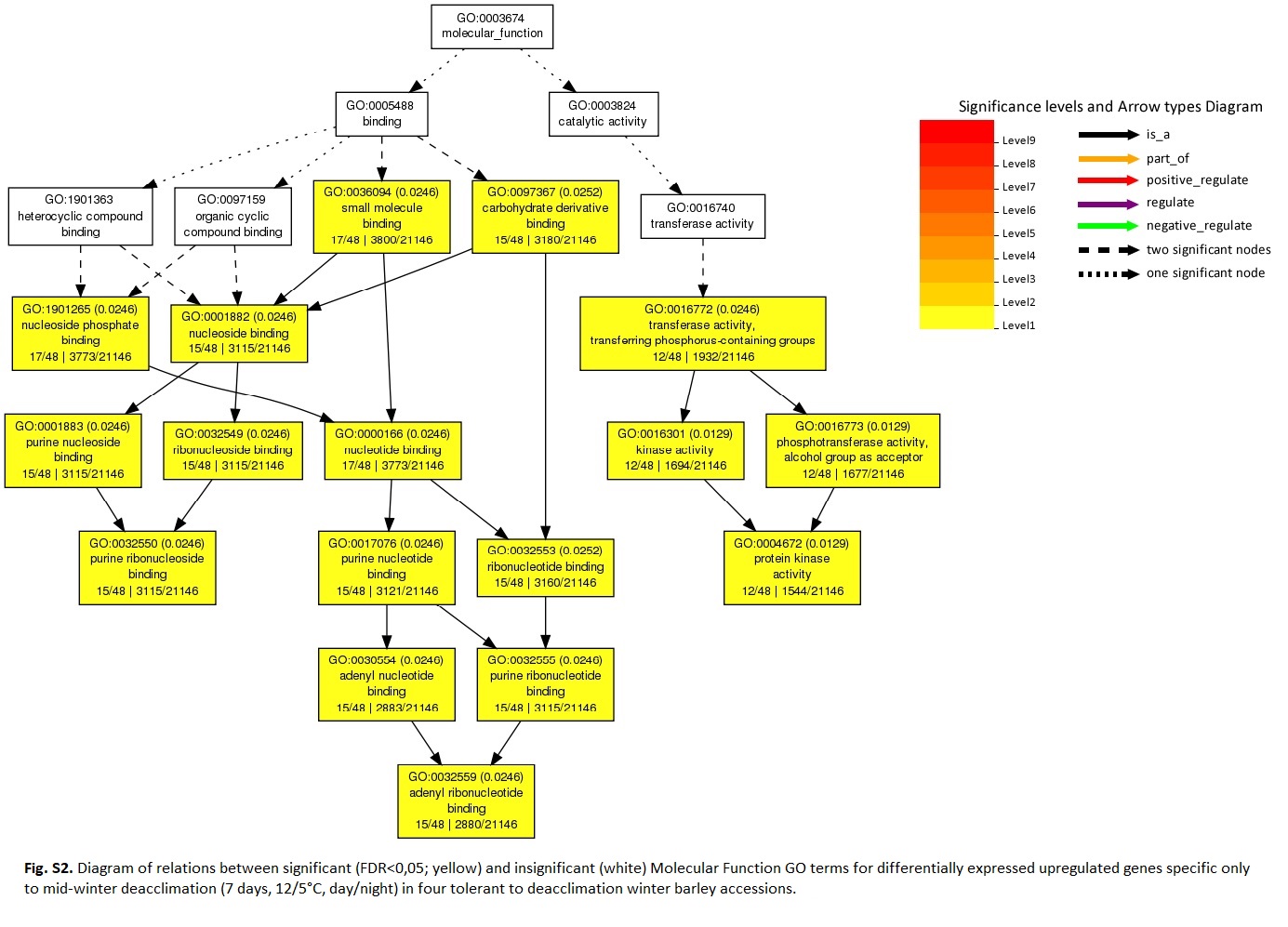

Supplement: Supplementary file 1 [file ijms-22-01057-s001.zip › supplementary/Figure S2.jpg]

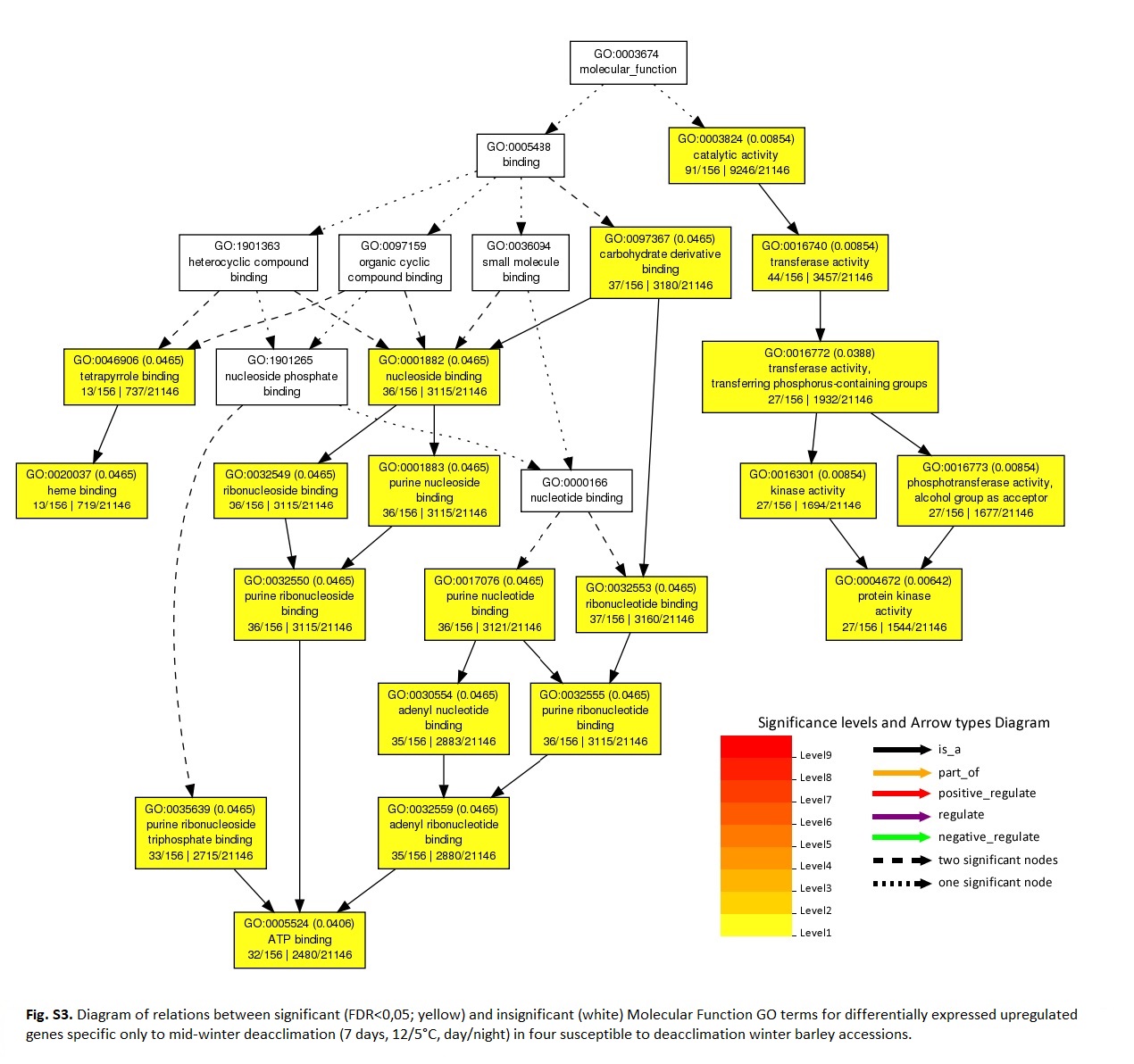

Supplement: Supplementary file 1 [file ijms-22-01057-s001.zip › supplementary/Figure S3.jpg]

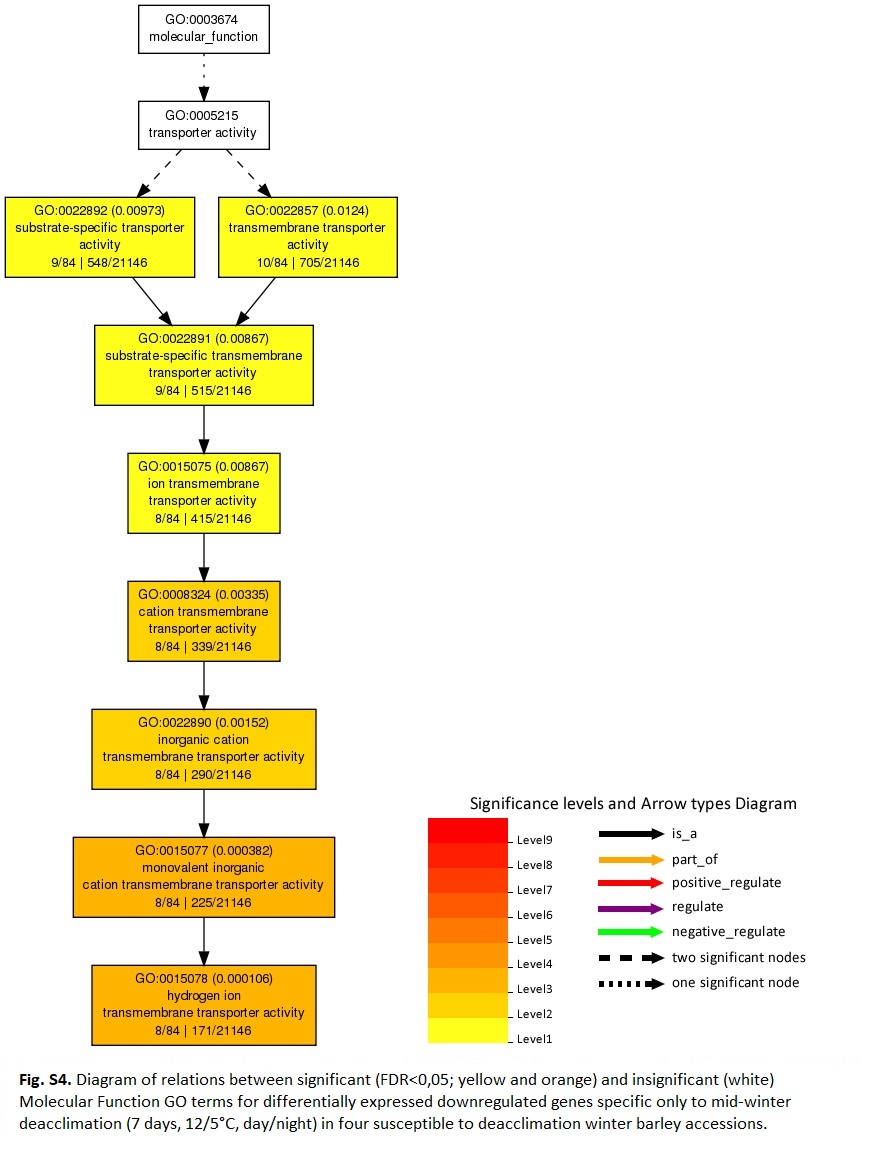

Supplement: Supplementary file 1 [file ijms-22-01057-s001.zip › supplementary/Figure S4.jpg]

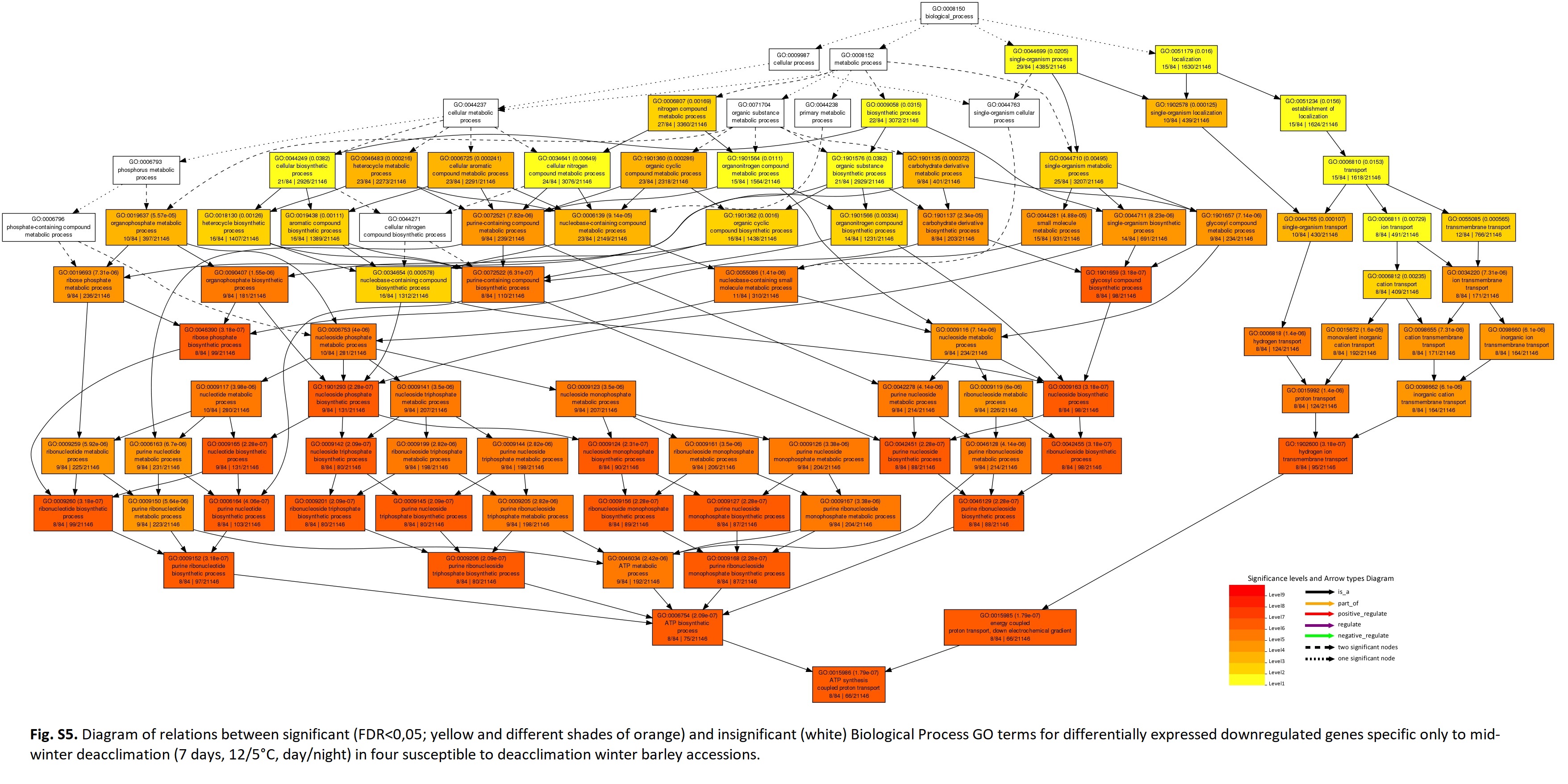

Supplement: Supplementary file 1 [file ijms-22-01057-s001.zip › supplementary/Figure S5.jpg]

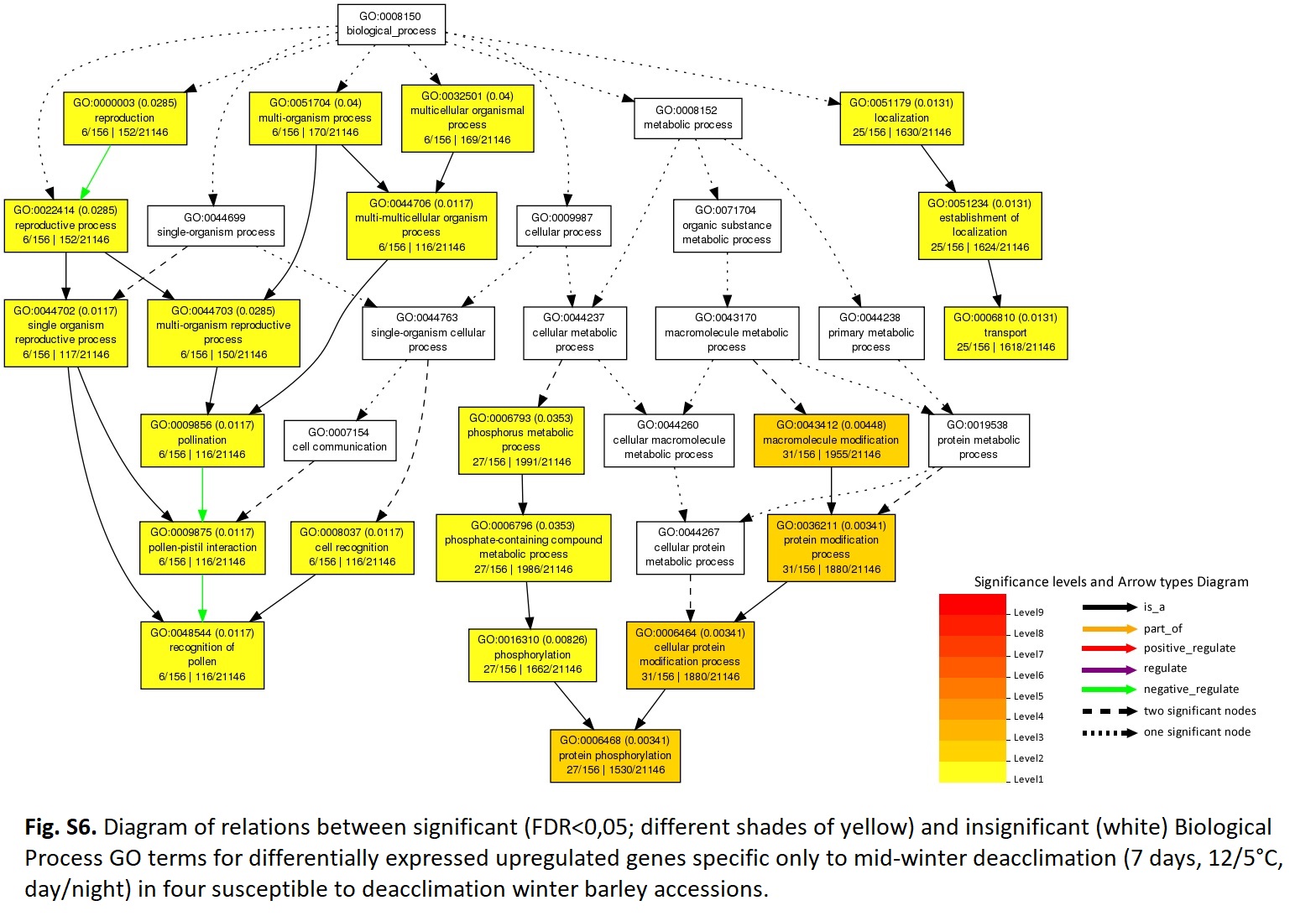

Supplement: Supplementary file 1 [file ijms-22-01057-s001.zip › supplementary/Figure S6.jpg]
